# Progressive Dysphagia Revealing Oculopharyngeal Muscular Dystrophy: A Diagnostic Challenge

**DOI:** 10.7759/cureus.117576

**Published:** 2026-10-07

**Authors:** Stuti Dalal, Jenny Jan, Katarina Greer

**Affiliations:** 1 Internal Medicine, Case Western Reserve University, Cleveland, USA; 2 Gastroenterology, Cone Health LeBauer Gastroenterology, Greensboro, USA; 3 Gastroenterology, Louis Stokes Cleveland Veterans Affairs Medical Center, Cleveland, USA

**Keywords:** dysphagia, gastroenterology, genetic, myopathy, ptosis

## Abstract

Oculopharyngeal muscular dystrophy (OPMD) is a rare, late-onset myopathy often presenting with progressive dysphagia and delayed diagnosis. We report a 69-year-old woman with worsening dysphagia, ptosis, and proximal muscle weakness who underwent an unrevealing gastrointestinal evaluation, including upper endoscopy. Videofluoroscopic swallow evaluation (VFSE) demonstrated pharyngeal dysphagia. A strong family history prompted genetic testing, confirming a poly(A) binding protein nuclear 1 (PABPN1) trinucleotide repeat expansion consistent with autosomal dominant OPMD.

This case highlights the importance of recognizing neuromuscular causes of dysphagia, particularly when structural evaluations are negative. Early identification can reduce diagnostic delay and allow appropriate supportive management, improving quality of life and reducing complications.

## Introduction

Oculopharyngeal muscular dystrophy (OPMD) is a rare, late-onset myopathy characterized by progressive ocular and pharyngeal muscle weakness and caused by pathogenic expansions in the poly(A) binding protein nuclear 1 (PABPN1) gene. Dysphagia is an early and prominent symptom caused by progressive pharyngeal muscular dysfunction but frequently prompts extensive gastrointestinal evaluation that may be unrevealing and thus be associated with significant delays in diagnosis [1-3]. We present a patient with decades of neuromuscular symptoms and progressive dysphagia beginning in her late 50s who underwent an extensive, unrevealing gastrointestinal evaluation and was ultimately diagnosed with OPMD through genetic testing.

This case emphasizes the importance of recognizing accompanying neuromuscular findings and a suggestive family history in patients with otherwise unexplained oropharyngeal dysphagia, which should prompt consideration of an inherited neuromuscular disorder and appropriate genetic testing to allow for multidisciplinary care to improve the patient’s quality of life.

## Case presentation

A 69-year-old female with a significant past medical history of type 2 diabetes and osteoarthritis presented with progressive dysphagia and weakness.

Per the patient, she developed bilateral ptosis in her early teens, requiring blepharoplasty, a history that was consistently documented. This recurred around age 45, requiring repeat blepharoplasty, at which point she also experienced proximal muscle weakness, worse in the hips than the shoulders. In her late 50s, she developed progressive dysphagia to both solids and liquids, which she described as moderately to severely distressing. The patient had multiple family members with similar problems: her mother had ptosis, her sister had dysphagia, a maternal uncle was diagnosed with muscular dystrophy, and multiple first-degree cousins were diagnosed with ptosis and/or dysphagia (Figure 1).

**Figure 1:**
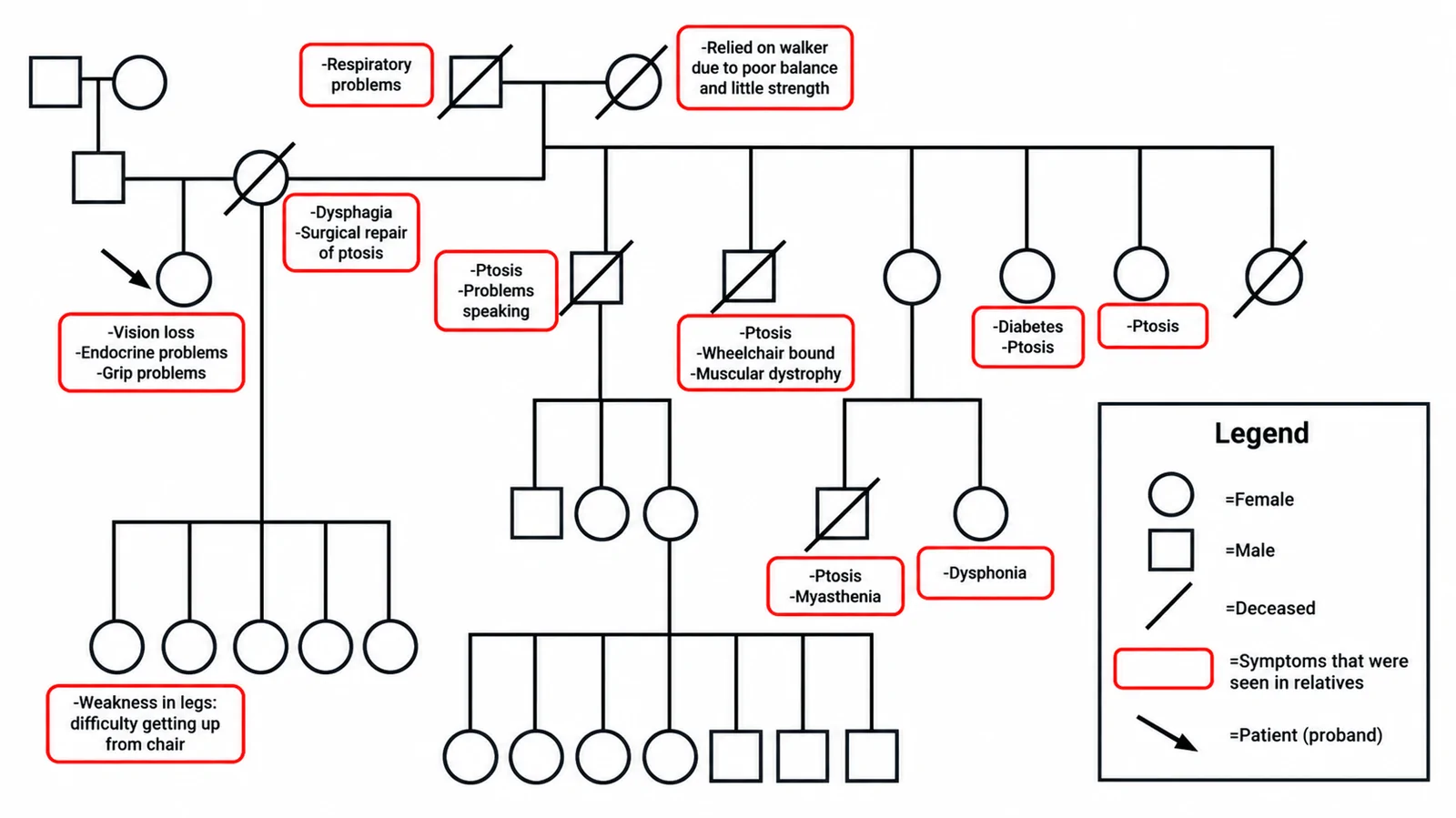
Family tree showing muscular manifestations in different family members as reported by the proband

On physical examination, the patient had bilateral ptosis and lower lid lag with weak eye closure. She had 2/5 strength in the upper extremities (deltoids and biceps) and 3/5 strength for hip flexion. She notably required significant arm effort to rise from a seated position. Abdominal examination was normal.

She underwent diagnostic evaluations over the years, including cross-sectional imaging and upper endoscopy, that did not reveal any structural causes of her dysphagia. Videofluoroscopic swallow evaluations (VFSE) performed eight years apart demonstrated reduced pharyngeal constriction and reduced base-of-tongue retraction, resulting in post-swallow pharyngeal residue. She also underwent high-resolution esophageal manometry (HREM), which demonstrated a decreased upper esophageal sphincter pressure of 22.1 mmHg (normal range, 34-104 mmHg), a distal contractile integral (DCI) of 5003.6 mmHg·s·cm (normal range, 500-5000), an elevated mean upper esophageal sphincter residual pressure of 15.0 mmHg (normal upper limit, 12.0 mmHg), a mildly increased lower esophageal sphincter resting pressure of 52.9 mmHg (upper limit of normal, 43 mmHg), and a normal integrated relaxation pressure of 10.6 mmHg (Figure 2). Despite extensive diagnostic workup, she still did not have a unifying diagnosis for her symptoms.

**Figure 2:**
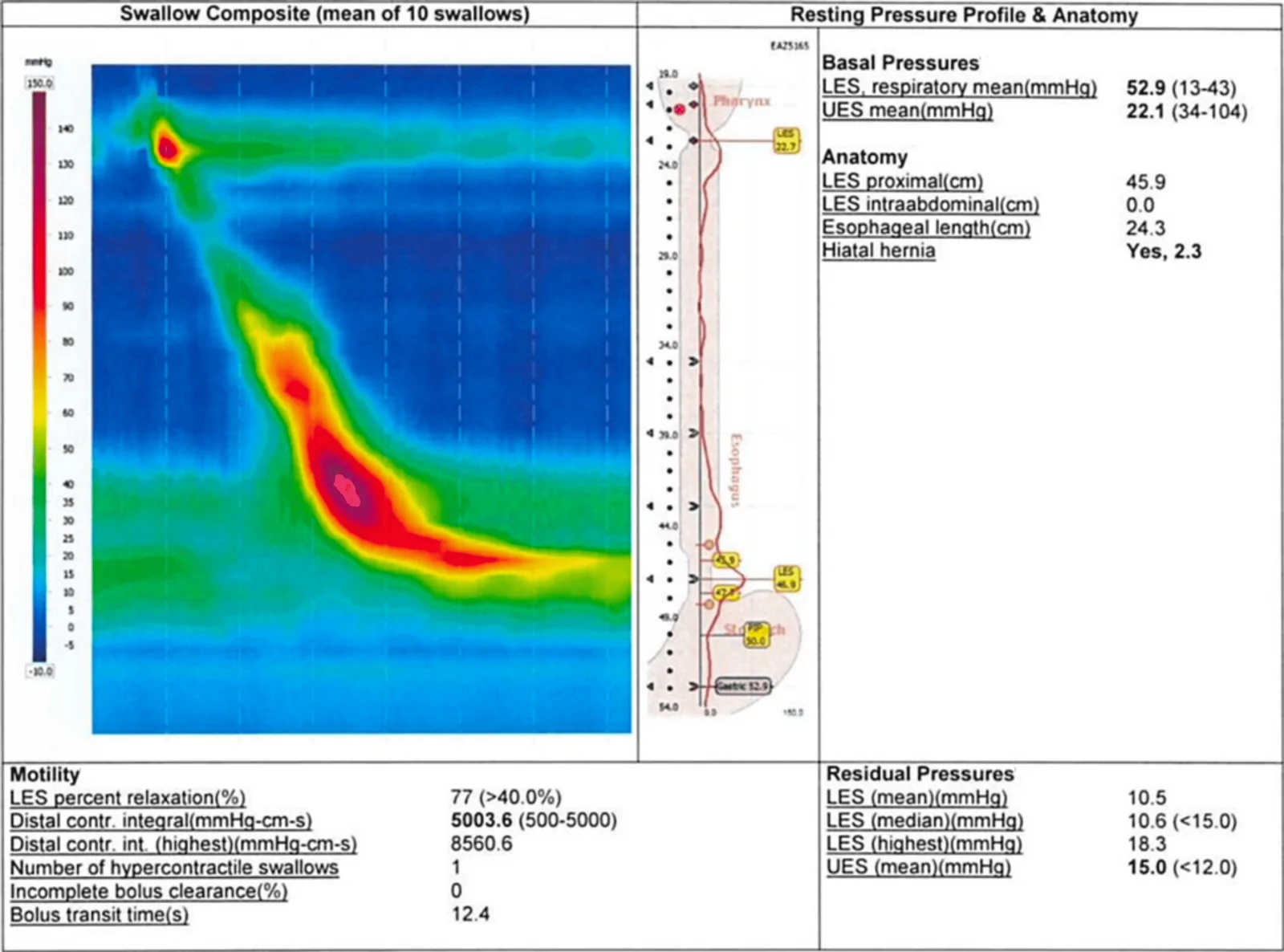
Manometry results Manometry results showing decreased resting upper esophageal sphincter (UES) pressure of 22.1 mmHg (normal range, 34-104 mmHg), elevated mean UES residual pressure of 15.0 mmHg (normal upper limit, 12.0 mmHg), a distal contractile integral (DCI) of 5003.6 mmHg·s·cm (normal range, 500-5000), a mildly increased lower esophageal sphincter (LES) resting pressure of 52.9 mmHg (upper limit of normal, 43 mmHg), and normal integrated relaxation pressure of 10.6 mmHg.

At the patient’s request, considering her extensive family history of ptosis and dysphagia, along with her own recurrent symptoms with no structural causes found, genetic testing was performed. The differential at this time included other neuromuscular etiologies, such as myasthenia gravis, inflammatory myopathies, myotonic dystrophies, or other inherited disorders. Genetic testing revealed an expansion of the guanine-cytosine-guanine (GCG) trinucleotide repeat in exon 1 of the PABPN1 gene, as well as 10 GCN and 14 GCN repeat alleles. The noted findings were consistent with the autosomal dominant form of OPMD [2]. This formal diagnosis allowed her to establish care with neuromuscular specialists and receive supportive services such as physical therapy and speech therapy. She was taught compensatory swallowing techniques and subsequently reported improvement in her swallowing symptoms at follow-up visits.

## Discussion

OPMD is a rare, late-onset myopathy that is frequently underrecognized, particularly when dysphagia is the presenting complaint. This case highlights several key challenges in the diagnosis of OPMD, including its insidious onset and the tendency for patients to undergo extensive but unrevealing structural evaluations prior to identification of a neuromuscular etiology.

Dysphagia in OPMD is primarily oropharyngeal and results from progressive weakness of the pharyngeal musculature, often involving the cricopharyngeal muscle. This leads to impaired bolus propulsion and incomplete upper esophageal sphincter relaxation, predisposing patients to choking and aspiration [4,5]. Gastroenterologists may become involved in the care of these patients early, given their presenting complaint of dysphagia. As seen in this case, structural evaluation, including upper endoscopy, did not reveal any anatomical or structural abnormalities. VFSE revealed mild-to-moderate pharyngeal nonspecific abnormalities. HREM shows a pattern that is also seen in other myopathies. This commonly includes reduced pharyngeal pressures, impaired relaxation and coordination of the pharyngoesophageal sphincter, and weak nonperistaltic contractions in the proximal esophagus [6]. Despite extensive diagnostic testing, there was still no unifying diagnosis for this patient. Nondiagnostic gastrointestinal testing is commonly described in OPMD; thus, diagnosis often hinges on recognition of associated neuromuscular features, such as ptosis and proximal muscle weakness, and a family history suggestive of hereditary neuromuscular disorders, prompting subsequent genetic testing for diagnosis [1,2]. Notably, the patient-reported onset of ptosis in early adolescence represents an atypically early presentation of OPMD, which more commonly manifests in a patient’s late 40s [7]. This unusually early presentation may have contributed to difficulty recognizing the later constellation of symptoms as manifestations of a single neuromuscular disorder.

The underlying pathophysiology of OPMD involves a trinucleotide repeat expansion in the PABPN1 gene, leading to intranuclear accumulation of abnormal protein aggregates and progressive muscle fiber dysfunction [2,8]. Although the disease course is typically slow, dysphagia can become the most clinically significant manifestation due to its impact on quality of life and risk of complications such as aspiration pneumonia and malnutrition [5,9]. While there is no cure for OPMD, early recognition remains critical, as it allows for timely implementation of supportive strategies, including dietary modification, swallow therapy, and, in select cases, procedural interventions such as cricopharyngeal myotomy or dilation, all of which can improve quality of life [10,11].

## Conclusions

In conclusion, OPMD remains an important but often overlooked cause of dysphagia. The constellation of oropharyngeal dysphagia with extraintestinal muscular weakness should raise suspicion for systemic neuromuscular disease. This case illustrates the potential for significant diagnostic delay in patients who undergo repeated gastrointestinal evaluations for dysphagia without a clear etiology. Increased awareness among gastroenterologists of the clinical features of OPMD, including ptosis, proximal muscle weakness, and a strong family history, may facilitate earlier diagnosis and improve patient outcomes.
